# Reply to: Alternative meta-analysis of behavioural interventions to promote action on climate change yields different conclusions

**DOI:** 10.1038/s41467-020-17614-6

**Published:** 2020-08-05

**Authors:** Claudia F. Nisa, Edyta M. Sasin, Daiane G. Faller, Birga M. Schumpe, Jocelyn J. Belanger

**Affiliations:** New York University Abu Dhabi, Saadiyat Island, PO Box 129188, Abu Dhabi, UAE

**Keywords:** Environmental social sciences, Scientific community, Social sciences

**Replying to** S. van der Linden & M. H. Goldberg *Nature Communications*10.1038/s41467-020-17613-7 (2020)

The point made by van der Linder and Goldberg^1^ about the impact of variance estimators is valid. Random-effects meta-analysis requires an estimation of heterogeneity, and which estimator is selected matters because it influences the calculation of the effect size^2,3^. However, there is no universally superior estimator. Whether an estimator is considered more or less biased in simulations studies depends on a variety of parameters such as, for instance, the number of studies included, the number of participants per study (*n*), and how much this *n* varies from study to study^3–5^.

The DerSimonian–Laird (DL) estimator is the most popular estimator in the literature^2–5^, implemented by default in multiple meta-analysis software^4^. Therefore, its use facilitates replication by a wide range of audiences with varying levels of technical expertise. Nonetheless, the DL estimator has been challenged when heterogeneity is moderate to high, when meta-analysis includes only a few studies, and/or when it examines binary outcomes^3,5,6^. Yet, DL has been shown to perform adequately when the number of studies in the meta-analysis is moderate to large (>~30), and estimating continuous outcomes^4,6,7^—consistent with our data.

Van der Linder and Goldberg note that the high heterogeneity associated with the overall estimate and some subgroups analyses requires additional attention. We follow van der Linder and Goldberg’s valid suggestion and present overall effect sizes comparing multiple estimators (Table 1). These authors also present some alternative estimates, but failed to discuss important nuances in their interpretation.

**Table 1:** Overall effect size per estimator.

Alternative estimators	Cohen’s *d*	95% CI	
Hunter–Schmidt^a^ (HS)	−0.058	−0.079	−0.037
Permutation Model^b^ (PE)	−0.093	−0.158	−0.055
DerSimonian–Laird^b,c^ (DL)	−0.093	−0.160	−0.055
Maximum-Likelihood^a^ (ML)	−0.193	−0.238	−0.148
Restricted Maximum-Likelihood^a^ (REML)	−0.194	−0.239	−0.148
Empirical Bayes^a^ [(EB) or Paule–Mandel]	−0.204	−0.251	−0.157
Sidik–Jonkman^a^ (SJ)	−0.219	−0.267	−0.170

The estimators compared alternatives available in STATA *Metaan* and R *Metagen*.

^a^R.

^b^STATA.

^c^DL estimate in R *d* = −0.093 [95% CI −0.113, −0.074].

Results show that the overall estimate ranges from *d* = −0.058 to *d* = −0.219, depending on the estimator used. The Hunter–Schmidt (HS) and the Sidik–Jonkman (SJ) estimators tend to report, respectively, negative and positive biased estimates compared to other estimators^4^. Maximum likelihood (ML) and Restricted Maximum Likelihood (REML) have good properties when heterogeneity is high and the size of studies varies considerably^5^, which applies well to our case. EB (equivalent to Paule–Mandel) has been recommended as an alternative to DL when the number of studies is small, and the heterogeneity is moderate to high^3–5^. Nevertheless, when meta-analysis combines small and large studies (as it is our case), EB tends to produce a positive bias compared with DL and REML^7^.

Table 1 shows that several estimators produce an overall effect size higher than what we reported. Does this mean our conclusions are wrong? No—our conclusions hold and here is why.

Firstly, our primary main conclusion is that behavioural interventions have a very small average effect size. This conclusion is grounded on Cohen’s *d* guidelines^8^—arbitrary to some extent but—followed in the absence of more objective standards. All effect sizes below *d* = 0.2 are interpreted as very small:^9^ if two groups’ means differ by <0.2 standard deviations, the difference between them is trivial, even if statistically significant^9,10^. The highest estimates within a variety of estimators reach, at best, the threshold of the *small effect* classification (which ranges from *d* = 0.20 to *d* = 0.49^9^). Moreover, the estimators that reach this threshold are associated with positive bias when there are large differences in study sizes (which is our case). We call to mind that Cohen’s *d* does not vary from 0 to 1, but from 0 to infinity, which puts in perspective the small differences between estimators. Moreover, these results can be compared to more intuitive measures of effect size. A Cohen’s *d* = 0.2 is equivalent to Cohen’s *r* = 0.1^10^, and it is difficult to argue that correlations *r* ≤ 0.1 could be interpreted as meaningful. Under several estimators, the probability of benefit^11^ doubles from 7% to 14%, equivalent to a probability of superiority^10^ changing from 53% to 56%, not much better than the flip of a coin.

Secondly, the idea that such small effects scaled at the population level can become meaningful is misleading. Interventions scaled up to the general population imply these will target a more heterogeneous set of individuals regarding their motivation to behave pro-environmentally, compared to the small-scale interventions where people self-select to participate^12^, page 4, 10)—which represent more than half our sample. This suggests that effect sizes would likely approach our estimates for naïve subjects (i.e., no self-selection) (*d* = −0.040 95% CI−0.103, −0.016).

Thirdly, van der Linder and Goldberg failed to note that random-effects meta-analysis pays more attention to small studies when pooling the overall effect^9^. Yet, small studies are prone to bias because small studies tend to be published only when reaching significance, and significance in small studies occurs when there are large effect sizes. This is a noteworthy concern because small studies (*n* ≤ 100) correspond to 57% of our total estimates (*k* = 82). We make this point in the original paper^12^, reporting a substantial small-studies bias^13^ and showing that an analysis restricted to the more robust samples (moderate and large studies) produces overall effect sizes well below *d* = 0.2. Table 2 shows that this conclusion holds across estimators.

**Table 2:** Sensitivity analyses per moderator with different estimators (Cohen’s *d*).

	DL	ML	REML	EB
Small studies (*n* ≤ 100) (*k* = 82)	−0.334	−0.335	−0.335	−0.334
Medium studies (100 < *n* < 500) (*k* = 45)	−0.137	−0.135	−0.135	−0.143
Large studies (≥500) (*k* = 17)	−0.028	−0.028	−0.028	−0.030
Information (*k* = 53)	−0.048	−0.036	−0.068	−0.083
Nudges (*k* = 11)	−0.352	−0.352	−0.352	−0.352
Social norms (*k* = 32)	−0.077	−0.191	−0.193	−0.198

Lastly, our secondary main conclusions also hold. We discouraged the isolated use of information-based interventions and its very small effect size (below *d* = 0.1) holds across estimators. We encouraged the use of nudges (firstly) and social comparison (secondly). The estimates for nudges hold across estimators ((*I*^2^ = 0). These results hold across estimators due to low between-studies heterogeneity. In the case of social norms, there is high between-studies heterogeneity (*I*^2^ = 72.2%), generating variation between estimators. But high heterogeneity often originates from substantial differences in sample sizes between the pooled studies^14^. And social norms are another good example of a strong statistically significant difference between small studies (*d* = −0.387, *n* ≤ 100, *k* = 13), and medium/large studies (*d* = −0.036, *n* > 100, *k* = 19) (meta-regression *t*-test = 5.21 *p* < 0.001).

Fundamentally, in case of high heterogeneity, DL provided more conservative estimates compared with other good alternatives, but DL estimates leaned closer to the estimates that would result from restricting the analysis to more robust samples, larger and naïve, less likely to be biased.

In summary, our conclusions hold because most overall effect sizes produced by different estimators are still interpretable as very small or, at best, borderline small. Furthermore, there is a substantial small-studies bias that pushes many estimates up. Notwithstanding, discussions about interventions and their (accurate) effect sizes are imperative and timely. The U.N. declared the next 10 years as the Decade of Action for climate change to deliver on the 2030 promises^15^, motivated by the awareness that action is not advancing at the speed or scale required, and calling for interventions to step-up their impact.

Stating that effect-sizes in psychology are known to be small should not be used as a justification to inflate the meaningfulness of (very) small effects. A thoughtful debate beyond statistical significance is long overdue to make psychological and behavioural science more relevant to intervention and policy-making.

## Data Availability

We provide a Source Data File that is publicly available at Figshare [10.6084/m9.figshare.9641999].

## Code availability

All commands used in STATA are publicly available here: metaan https://www.stata-journal.com/article.html?article = st0201[*metaan smd se, dl* [OR *pe* OR *ml*].These analyses can be replicated in R using metagen or rma, and these R codes are publicly available at Figshare [10.6084/m9.figshare.9641999]. The code is the same has presented at Figshare; the only change to be made between estimators is in “method.tau = "DL", which needs to be replaced by alternatives initials e.g., ML, REML, EB.
